# Effect of robotic arm-assisted bi-unicompartmental knee arthroplasty versus total knee arthroplasty on objectively measured physical activity: secondary outcomes of a randomized controlled trial

**DOI:** 10.1302/2046-3758.159.BJR-2026-0018.R1

**Published:** 2026-09-10

**Authors:** Sree Kanakala, Miloš Brkljac, Matthew Banger, James Doonan, Søren Brage, Mark Blyth, Philip Rowe, Bryn Jones, Angus MacLean, Tim Lindsay

**Affiliations:** 1 School of Medicine, Imperial College London, London, UK; 2 MSk Lab, Imperial College London, London, UK; 3 Department of Trauma and Orthopaedics, NHS Greater Glasgow and Clyde, Glasgow, UK; 4 MRC Epidemiology Unit, University of Cambridge, Cambridge, UK; 5 Department of Biomedical Engineering, University of Strathclyde, Glasgow, UK

**Keywords:** Bi-unicompartmental knee arthroplasty, Total knee arthroplasty, Physical activity, randomized controlled trial, unicompartmental knee arthroplasty, total knee arthroplasty (TKA), robotic arm, patient-reported outcome measures (PROMs), accelerometers, arthroplasty, thigh, hip and knee arthroplasty, knees

## Abstract

**Aims:**

Bi-unicompartmental knee arthroplasty (Bi-UKA) is a bone-conserving, cruciate-sparing alternative to total knee arthroplasty (TKA). Patient-reported outcome measures (PROMs) do not differ between procedures. Conflicting data exist on gait preservation, and little is known about physical activity (PA) outcomes. PA is understudied in arthroplasty, despite its links with all-cause mortality. We compared PA volume and intensity in patients randomized to Bi-UKA or TKA.

**Methods:**

We analyzed 38 TKA and 31 Bi-UKA patients from a prospective randomized controlled trial. A triaxial accelerometer was worn on the surgical thigh for seven days, with a three-day minimum wear criterion. Data were collected preoperatively, and at one and two years postoperatively. Outcomes were PA energy expenditure (PAEE), moderate-to-vigorous intensity PA (MVPA), light-intensity PA (LPA), and sedentary time (ST), plus proportional PAEE by intensity. Group differences were assessed with mixed-effects models including baseline PA and biphasic gait as covariates. Spearman’s rank correlation between PROMs and PA was performed.

**Results:**

Preoperative characteristics were comparable. Activity levels were higher following surgery, but postoperative differences in PAEE, MVPA, LPA, or ST between groups failed to reach statistical significance. Baseline PAEE (*β* =+ 0.27, p = 0.037) and MVPA (*β* =+ 0.25, p = 0.024) were associated with improvements in postoperative activity. Postoperative correlations between PROMs and objective PA were weak across groups, and appeared to diminish over time.

**Conclusion:**

Although free-living PA improved following Bi-UKA and TKA, differences between groups failed to reach statistical significance. Across both procedures, habitual and behavioural factors appear to be stronger determinants of postoperative PA than surgical procedure, as reflected by the limited correlation between PROMs and objective PA. Larger, long-term randomized studies are needed to confirm these findings.

Cite this article: *Bone Joint Res* 2026;15(9):1136–1146.

## Article focus

- We aimed to compare free-living physical activity (PA) after bi-unicompartmental knee arthroplasty (Bi-UKA) versus total knee arthroplasty (TKA) using thigh-worn accelerometry.
- As a secondary aim, we explored the link between patient-reported outcome measures (PROMs) and free-living PA following knee arthroplasty.

## Key messages

- Differences in free-living PA between Bi-UKA and TKA did not reach statistical significance.
- Preoperative PA levels were stronger determinants of PA than surgical procedure.
- PROMs weakly correlated with objective PA, suggesting there may only be a weak relationship between self-reported and objective function.

## Strengths and limitations

- This randomized controlled trial provides a novel comparison of PA outcomes between TKA and Bi-UKA using a robust processing approach.
- The study was not powered to detect differences in PA between groups, and therefore these results should be interpreted with caution.
- The limited sample size limits the generalizability of our findings.

## Introduction

Total knee arthroplasty (TKA) is the most commonly performed intervention for end-stage osteoarthritis (OA) of the knee.^1-4^ While TKA is considered the gold standard of treatment, the majority of gonarthrosis only affects one or two knee compartments.^5^ TKA has been shown to routinely sacrifice healthy structures, such as the ACL, in up to 78% of cases.^6^ With dissatisfaction rates ranging between 15% and 30% following TKA,^7^ there is a need for a safe alternative that preserves tissue and offers a return to high-level function. Bi-unicompartmental knee arthroplasty (Bi-UKA) is a bone-conserving, tissue-sparing option for patients with combined medial and lateral gonarthrosis. By selectively resurfacing diseased compartments, Bi-UKA conserves all ligamentous structures, thus better preserving native knee kinematics such as femoral rollback and the screw-home mechanism.^8^ Previously published results have demonstrated favourable alignment, gait characteristics, and patient-reported outcome measures (PROMs) following Bi-UKA compared with TKA.^9,10^ However, it is unknown whether any potential advantages conferred by Bi-UKA translate into increased activity in free-living conditions.

Physical activity (PA) is an important outcome worthy of study. In a large population-based study, Strain et al^11^ showed that both volume and intensity of PA were independently associated with all-cause mortality. More recently, a UK Biobank analysis found that accelerometer-derived PA predicts mortality following hip and knee arthroplasty.^12^ For patients undergoing knee arthroplasty, postoperative PA is therefore important for long-term health and function. Despite its benefits, the current evidence on PA following knee arthroplasty is largely confined to TKA.^13^ It is not yet known whether tissue-preserving procedures, such as Bi-UKA, yield superior free-living activity outcomes compared with TKA.

PROMs are routinely used in the assessment of arthroplasty and are collected by numerous national registries.^1,14^ However, traditional PROMs, such as the Oxford Knee Score (OKS),^15,16^ may demonstrate a ceiling effect, and therefore do not always distinguish between high-functioning patients.^17^ Wearable technology, such as research-grade accelerometers, can quantify PA volume and intensity in free-living conditions following knee arthroplasty.^18,19^ Wearables may help address the ceiling effects seen with traditional PROMs and allow a more detailed assessment of postoperative function than PROMs alone.^20^

To our knowledge, the present study is the first to compare accelerometer-measured PA levels between robotic Bi-UKA and manual TKA. Our work forms part of the secondary outcome analysis of the Total versus Robotic bi-UniCompartmental Knee (TRUCK) Trial. We assessed changes in PA volume and intensity from preoperative baseline to one and two years postoperatively. We hypothesized that Bi-UKA would lead to significantly higher levels of PA than TKA at one and two years postoperatively. The null hypothesis was that there would be no difference in PA outcomes between TKA and Bi-UKA at these specified timepoints.

## Methods

### Study setting and participants

This study used data from the TRUCK Trial, a prospective, randomized controlled trial (RCT) comparing manual TKA and robotic-assisted Bi-UKA. Patients were eligible to participate in the trial if they had medial and lateral compartment OA suitable for unconstrained TKA, and if their cruciate and collateral ligaments were preserved. Patients with a varus/valgus deformity exceeding 15°, fixed flexion contracture deformity exceeding 10°, patellofemoral OA above Kellgren-Lawrence grade III,^21^ isolated unicompartmental disease amenable to UKA, or any inflammatory arthropathy were excluded.^9^ TKA patients received fixed-bearing, cruciate-sacrificing Zimmer NexGen LPS implants (Zimmer Biomet, USA). Bi-UKA patients received two unicondylar fixed-bearing MAKO Restoris MCK implants (Stryker, USA) placed medially and laterally, guided by preoperative CT planning and robotic arm assistance (MAKO RIO System).^22^ A single medial parapatellar approach was used in both groups.

**Fig. 1:**
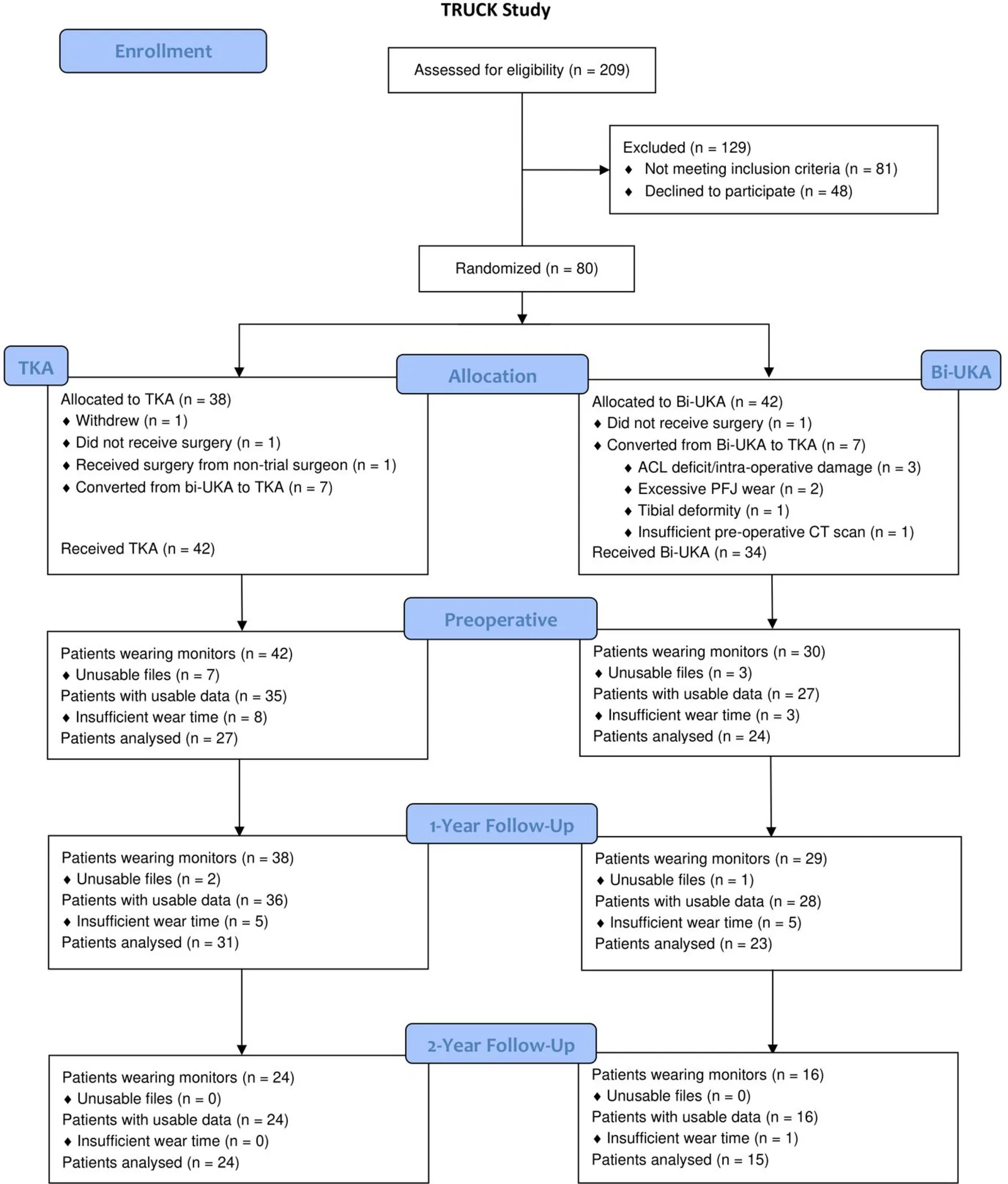
CONSORT diagram showing patient flow and valid physical activity data in the TRUCK study. ACL, anterior cruciate ligament; Bi-UKA, bi-unicompartmental knee arthroplasty; PFJ, patellofemoral joint; TKA, total knee arthroplasty.

Of the 80 patients randomized in the TRUCK trial, 76 underwent surgery (TKA = 42, Bi-UKA = 34) and were eligible for analysis (Figure 1). One patient had missing preoperative accelerometer data and did not attend postoperative follow-up, and therefore did not contribute any PA data. A total of 75 patients provided accelerometer data (TKA = 42, Bi-UKA = 33). After applying wear-time criteria and excluding unusable files, 69 patients (TKA = 38, Bi-UKA = 31) were included in the present analysis. Participants were included if they contributed valid PA data at any timepoint. Participant flow and the availability of valid PA data at each timepoint are shown in the CONSORT flow diagram (Figure 1).

### Patient characteristics

Preoperative characteristics were similar between groups for all participants contributing valid PA data (n = 69; TKA = 38, Bi-UKA = 31) (Table I). Mean age was 70.3 years (SD 7.0) in the TKA group and 69.0 years (SD 7.8) in the Bi-UKA group. Mean BMI was 32.4 kg/m² (SD 5.4) and 32.2 kg/m² (SD 6.8) in the TKA and Bi-UKA groups, respectively. No differences in preoperative characteristics were observed between groups for timepoint-specific analyses (Supplementary Table 1). Overall accelerometer compliance was 80%, with 12% of recordings excluded due to insufficient wear time and 7% of recordings excluded due to unusable data (Figure 1).

**Table I.** Preoperative characteristics of total knee arthroplasty (TKA) and bi-unicompartmental knee arthroplasty (Bi-UKA) patients contributing valid physical activity data at any timepoint in the study.

Characteristic	TKA	Bi-UKA	p-value
N	38	31	N/A
Mean age, yrs (SD)	70.3 (7.0)	69.0 (7.8)	0.476*
Operated side (right/left), n	14/24	15/16	0.334†
Sex (male/female), n	19/19	17/14	0.689†
Mean height, cm (SD)	163 (11)	162 (11)	0.841*
Mean weight, kg (SD)	85.9 (16.0)	84.1 (14.5)	0.615*
Mean BMI, kg/m^2^ (SD)	32.4 (5.4)	32.2 (6.8)	0.907*

Values reflect preoperative measurements only.

* Welch’s *t*-test.

† Chi-squared test.

N/A, not applicable.

### Physical activity measurement

Participants were asked to wear an ActivPAL4 triaxial accelerometer (PAL Technologies, UK) continuously for seven days and nights at three timepoints: preoperatively (mean 2.3 months before surgery (SD 1.9)), and at one year (mean 12.8 months (SD 1.8)) and two years (mean 28.2 months (SD 5.4)) postoperatively. Monitors recorded raw triaxial acceleration at a sampling rate of 20 Hz with a dynamic range of ± 4 g. The monitor was fitted to the anterior midline of the surgical mid-thigh using a nitrile sleeve and waterproof adhesive dressing (3 M Tegaderm; Solventum, UK). Participants did not need to remove the device when showering.^23^

### Accelerometer data processing

Following download of the monitors’ data, raw triaxial data were exported using MATLAB (Mathworks, USA). Data were calibrated to local gravity and vector magnitude (VM) calculated from accelerations in the three orthogonal axes.^24,25^ Euclidean Norm Minus One (ENMO) was derived by subtracting 1 g from vector magnitude to remove the gravity component of the acceleration signal and isolate the activity-related signal, expressed in milli-g (mg).^26^ Negative values were truncated to zero.^26^

All participant files had a post-calibration error of < 0.02 g, and were hence used in subsequent analysis.^27^ The package *GGIR* (v. 3.0-3) was used to process the data.^28^ Periods of non-wear were identified as time segments of low movement variability relative to sensor noise levels; axis acceleration below 13 mg for thigh acceleration.^28^ Data were collapsed to five-second epochs, and average ENMO was calculated over all available data normalized per 24-hour cycles (diurnally balanced), with invalid data imputed by the average at similar timepoints on different days of the week.

All data were plotted and manually verified. An example of the raw signals and subsequently derived ENMO across a 24-hour period for one participant is displayed in Supplementary Figure 1. Inclusion criteria for analysis specified total wear time > 72 hours,^29,30^ and ≥ three days wear time with ≥ ten hours wear per day, consistent with prior accelerometry studies.^31,32^ Accelerometer compliance was defined as the proportion of accelerometer datasets included in the analysis.

### Outcomes

Accelerometry outcomes included overall movement volume (average ENMO (mg)), and time spent sedentary (ST, min/day), in light-intensity PA (LPA, min/day) and moderate-vigorous-intensity physical activity (MVPA, min/day). Cut-points of 30 mg and 105 mg were applied for ST (< 30 mg), LPA (30 to 105 mg), and MVPA (> 105 mg) (Supplementary Figure 2).^33^ MVPA was defined as at least one minute where 80% or more of the five-second epochs met the 105 mg MVPA threshold.^34^ This definition, as distinct from total MVPA, shows stronger and more consistent positive health correlations in osteoarthritic older adults, irrespective of the cut-points used.^35,36^ PA energy expenditure (PAEE) was calculated using linear ENMO equations validated against doubly labelled water.^37^ PAEE and MVPA were treated as the primary PA outcomes, as they reflect daily activity volume and high-intensity activity. Proportion of PAEE accumulated through MVPA, LPA, and ST were calculated as the proportion of energy expenditure from each intensity relative to the total activity energy expenditure from all activity.

PROMs (OKS, New Knee Society Score (NKSS),^38^ UCLA Activity Scale)^39^ were collected at each timepoint as described previously.^40^ PROM data are reported only for participants who contributed valid PA data. A separate publication reports PROM outcomes for the full trial cohort.^41^ Spearman correlations between PROMs and PA outcomes were calculated within each group and timepoint.

### Statistical analysis

The TRUCK trial was powered to detect a difference in the proportion of patients demonstrating a biphasic sagittal knee moment curve during level walking at one year postoperatively. The study was not powered for PA outcomes. We used the one-year PAEE data to determine the minimum detectable difference (MDD) between groups. At 80% power with an α of 0.05, the MDD in PAEE at one year was 3.44 kJ day^-1^kg^-1^. Smaller differences between groups cannot be excluded.

All statistical analyses were performed using R (v. 4.4.3, R Foundation for Statistical Computing, Austria). Descriptive characteristics are presented as mean (SD) for parametric data and median (IQR) for non-parametric data. Categorical data are presented as n (%). The Welch *t*-test was used for parametric data. Categorical data were analyzed by the chi-squared test.

A multilevel mixed effects model of postoperative PA was created using *lme4* and *lmertest* packages (Equation 1).^42^ Sociodemographic covariates (age, sex, BMI) were not included, as preoperative characteristics were comparable between groups following randomization. The primary RCT outcome, biphasic gait, was included as a covariate. Subgroup analyses were not performed due to limited sample size. Mixed-effects analysis of variance (ANOVA) was performed at a 5% significance level to compare trial arms.

Equation 1. Multilevel mixed effects model of postoperative activity. Model accounts for biphasic gait and preoperative activity:

*Postoperative PA = Preoperative PA + Biphasic Gait + Group + Time + Group x Time*

## Results

### Physical activity outcomes

**Fig. 2:**
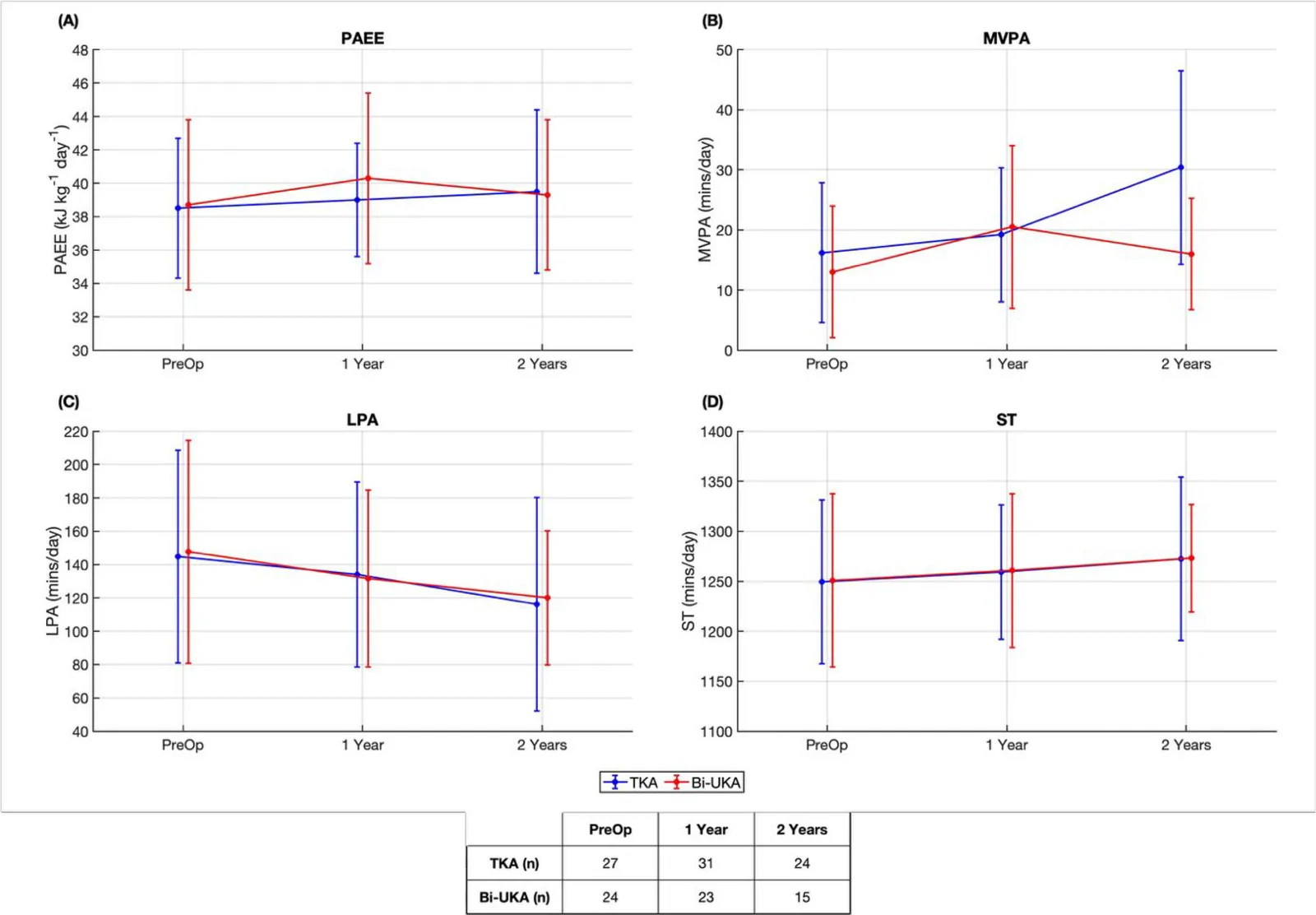
Line graphs showing a) physical activity energy expenditure (PAEE), b) moderate-to-vigorous physical activity (MVPA), c) light PA (LPA), and d) sedentary time (ST) in total knee arthroplasty (TKA) and bi-unicompartmental knee arthroplasty (Bi-UKA) patients. PAEE, LPA, and ST are presented as mean (SD), and MVPA as median (IQR). Data are displayed at three timepoints: preoperative baseline, and one and two years postoperatively.

**Fig. 3:**
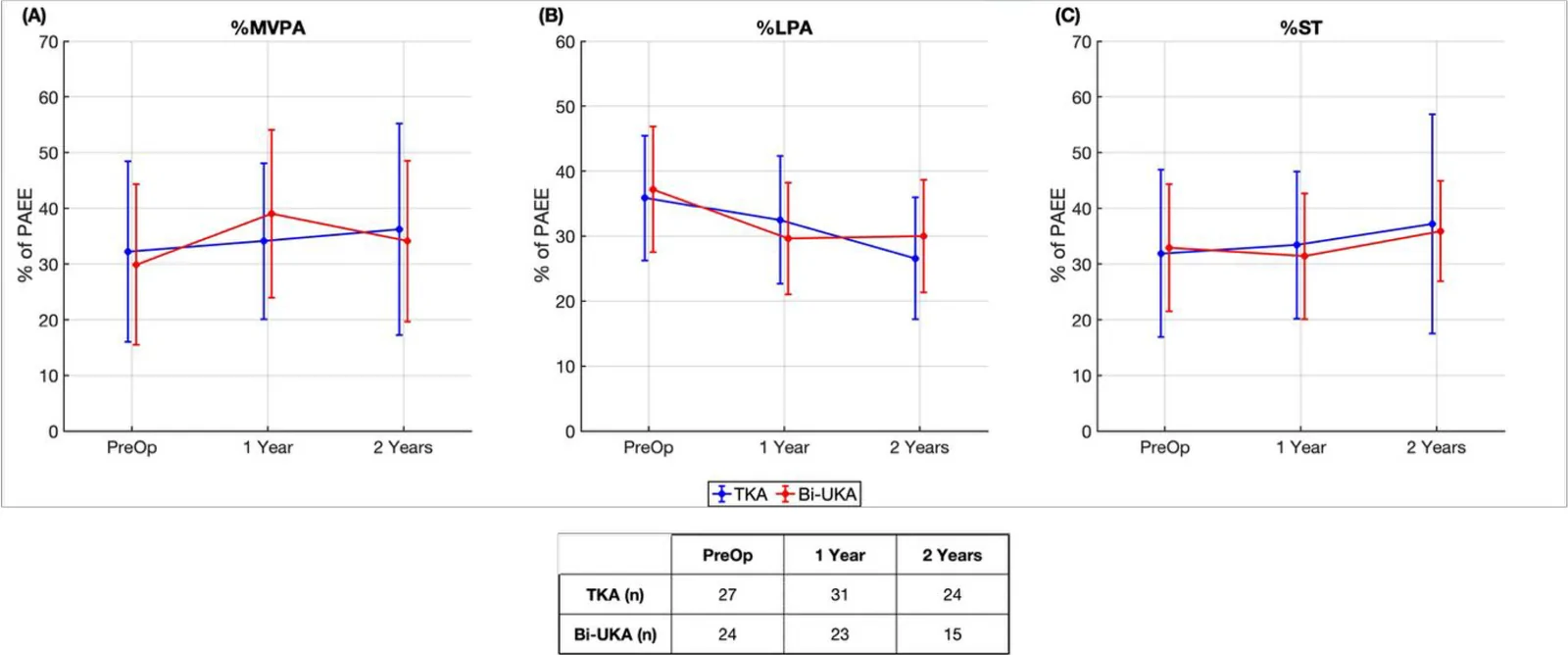
Line graphs showing percentage contribution of a) moderate-to-vigorous physical activity (MVPA), b) light PA (LPA), and c) sedentary time (ST) to PA energy expenditure (PAEE) over time in total knee arthroplasty (TKA) and bi-unicompartmental knee arthroplasty (Bi-UKA) patients (mean (SD) at preoperative baseline and one- and two-year follow-up).

**Fig. 4:**
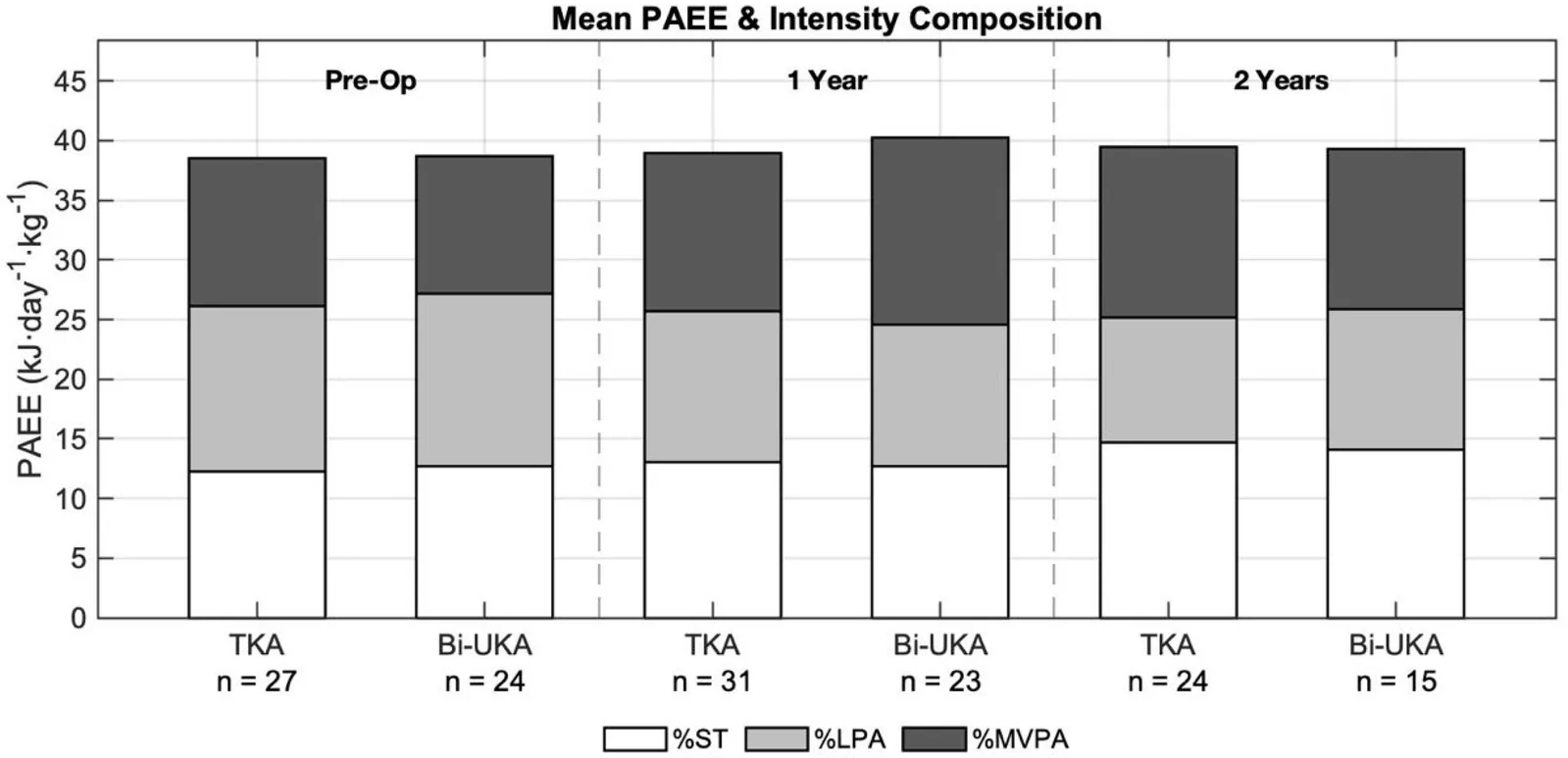
Mean physical activity energy expenditure (PAEE) and its intensity composition in total knee arthroplasty (TKA) and bi-unicompartmental knee arthroplasty (Bi-UKA) patients at preoperative baseline, and one and two years after surgery. Bars show mean PAEE (kJ day⁻¹ kg⁻¹) divided into the proportional energy contributions of sedentary time (ST), light physical activity (LPA), and moderate-to-vigorous physical activity (MVPA).

Preoperative PA levels were comparable between groups (Table II). Differences between groups in daily PA volume (PAEE) or PA intensity (MVPA, LPA, ST) did not reach statistical significance at one and two years postoperatively (Table II, Figure 2). Similar findings were observed for the proportion of daily activity energy expenditure derived from each activity intensity (Table II, Figure 3 and Figure 4). Descriptively, Bi-UKA showed a greater total activity volume and moderate-to-vigorous PA than TKA at one year, which regressed by two years (Figure 2). TKA showed a small rise in PAEE and MVPA across timepoints. Both groups showed decreases in light PA and increases in sedentary time over the two-year follow-up.

**Table II.** Descriptive statistics of objectively measured physical activity following total knee arthroplasty (TKA) and bi-unicompartmental knee arthroplasty (Bi-UKA).

Outcome	TKA			Bi-UKA		
	Preop (n = 27)	1 year postop (n = 31)	2 years postop (n = 24)	Preop (n = 24)	1 year postop (n = 23)	2 years postop (n = 15)
PAEE, kJ day^-1^kg^-1^	38.5 (4.2)	39.0 (3.4)	39.5 (4.9)	38.7 (5.1)	40.3 (5.1)	39.3 (4.5)
MVPA, mins/day	16.2 (9.8 to 33.1)	19.2 (11.0 to 33.3)	30.4 (8.9 to 41.1)	13.0 (6.3 to 28.2)	20.5 (11.6 to 38.7)	16.0 (9.4 to 27.9)
LPA, mins/day	144.8 (63.8)	134.0 (55.5)	116.2 (64.1)	147.7 (66.9)	131.6 (53.0)	120.0 (40.2)
ST, mins/day	1,249.5 (81.9)	1,259.2 (67.1)	1,272.3 (81.8)	1,250.7 (86.6)	1,260.6 (76.8)	1,273.2 (53.8)
%MVPA, %PAEE	32.2 (16.2)	34.1 (14.0)	36.2 (19.0)	29.9 (14.4)	39.0 (15.1)	34.1 (14.4)
%LPA, %PAEE	35.9 (9.6)	32.5 (9.8)	26.6 (9.4)	37.2 (9.7)	29.6 (8.6)	30.0 (8.7)
%ST, %PAEE	31.9 (15.0)	33.4 (13.2)	37.2 (19.7)	32.9 (11.4)	31.4 (11.3)	35.9 (9.0)

Values are shown at preoperative baseline, and one- and two-year follow-up for each procedure group. Sample sizes reflect the number of participants with valid accelerometer data at each timepoint. Values are presented as mean (SD) for normally distributed outcomes (PAEE, LPA, ST, %MVPA, %LPA, %ST) and median (IQR) for non-parametric outcomes (MVPA).

LPA, light physical activity; MVPA, moderate-to-vigorous physical activity; PAEE, physical activity energy expenditure; ST, sedentary time.

### Multilevel mixed effects analysis

The procedure performed did not significantly influence any PA outcome (Table III). Greater preoperative PA volume (β = + 0.27, p = 0.037) and moderate-to-vigorous activity levels (β = + 0.25, p = 0.024) were significantly associated with higher postoperative activity (Table III). Biphasic gait trended towards a positive association with postoperative PA, although this relationship did not reach statistical significance.

**Table III.** Results of multilevel mixed-effects models for postoperative physical activity (PA) outcomes.

Outcome	Estimate	SE	p-value
**PAEE, kJ day** ^**-1**^ **kg** ^**-1**^			
Intercept	27.71	4.86	< 0.001
Group	+ 0.88	1.33	0.508
Time	+ 1.02	1.00	0.316
Baseline PAEE	+ 0.27	0.13	0.037
Biphasic gait	+ 1.60	1.08	0.141
Group x time	-1.93	1.71	0.266
**MVPA, mins/day**			
Intercept	13.42	6.07	0.031
Group	+ 7.67	6.55	0.246
Time	+ 4.01	4.81	0.412
Baseline MVPA	+ 0.25	0.11	0.024
Biphasic gait	+ 6.78	5.26	0.202
Group x time	-12.66	8.30	0.136
**LPA, mins/day**			
Intercept	121.65	20.96	< 0.001
Group	-13.31	16.77	0.430
Time	-11.80	12.38	0.348
Baseline LPA	+ 0.01	0.11	0.905
Biphasic gait	+ 19.32	13.51	0.157
Group x time	+ 13.05	21.39	0.545
**ST, mins/day**			
Intercept	1,174.78	137.65	< 0.001
Group	+ 5.15	21.37	0.810
Time	+ 2.11	16.77	0.901
Baseline ST	+ 0.08	0.11	0.458
Biphasic gait	-32.57	17.32	0.065
Group x time	+ 7.98	28.61	0.782

Models were adjusted for baseline (preoperative) outcome values and biphasic gait, with a random intercept for patient ID. The intercept represents TKA at one year postoperatively. The ‘Group’ term represents the difference between Bi-UKA and TKA at one year. The ‘Time’ term represents within-group change from one to two years. The ‘Group x Time’ term represents any additional change in the Bi-UKA group from one to two years.

Bi-UKA, bi-unicompartmental knee arthroplasty; LPA, light physical activity; MVPA, moderate-to-vigorous physical activity; PAEE, physical activity energy expenditure; ST, sedentary time; TKA, total knee arthroplasty.

### Patient-reported outcome measures

PROMs improved from preoperative baseline in both groups (Table IV). At one and two years postoperatively, OKS, UCLA, and NKSS Knee and Function scores were comparable between groups (Table IV). Correlations between PROMs and objectively measured PA were weak and inconsistent (Supplementary Table 2).

**Table IV.** Patient-reported outcome measures (PROMs) for participants included in the physical activity analysis, presented by intervention group and timepoint (preoperative, and one and two years postoperatively).

Outcome	TKA			Bi-UKA		
	Preop (n = 26)	1 year postop (n = 31)	2 years postop (n = 22)	Preop (n = 24)	1 year postop (n = 23)	2 years postop (n = 14)
OKS	21.0 (16.2 to 25.8)	41.0 (34.2 to 45.8)*	44.0 (40.2 to 46.0)	19.0 (14.0 to 22.5)*	39.0 (37.5 to 42.5)	42.0 (35.2 to 46.5)
UCLA	3.0 (3.0 to 4.0)	5.0 (4.0 to 6.0)	6.0 (5.0 to 6.0)	3.5 (3.0 to 5.2)	6.0 (4.5 to 7.0)	6.0 (5.0 to 7.5)
NKSS Knee	41.5 (38.0 to 47.0)	52.0 (45.0 to 56.5)	52.0 (47.2 to 57.0)	39.5 (35.0 to 47.5)	48.0 (43.0 to 54.0)	55.5 (50.2 to 57.8)
NKSS Function	33.0 (22.0 to 42.2)	74.0 (56.5 to 82.0)	85.5 (63.8 to 108.2)	32.5 (22.5 to 43.5)	74.0 (58.5 to 83.5)	97.5 (89.0 to 100.8)

Data are reported as median (IQR).

* One participant had missing data for this PROM at this timepoint; n is therefore one lower for this outcome only. All other PROMs at this timepoint use the column n.

Bi-UKA, bi-unicompartmental knee arthroplasty; NKSS, New Knee Society Score; OKS, Oxford Knee Score; TKA, total knee arthroplasty; UCLA, University of California Los Angeles Activity Scale.

## Discussion

This secondary analysis of the TRUCK RCT provides the first report of accelerometer-derived PA following Bi-UKA. While previous studies have evaluated function, gait, and activity using PROMs and laboratory-based performance tests,^10,43^ the objective assessment of PA following Bi-UKA in a free-living environment remains limited. We hypothesized that Bi-UKA, a tissue-preserving procedure that better maintains native knee kinematics, would lead to greater postoperative PA volume and intensity compared to TKA. However, differences in PA outcomes between procedures did not reach statistical significance over two-year follow-up. Descriptively, Bi-UKA showed a small rise in daily physical activity volume (PAEE) and high-intensity activity (MVPA) at one year that was not sustained at two years. However, given the TRUCK trial was not powered to detect differences in PA outcomes, these findings should be interpreted with caution.

As objective PA data on Bi-UKA remain limited, UKA cohorts provide the closest available benchmark. The strength of comparisons across procedures is limited, as UKA and Bi-UKA are performed in patients with different distributions and severity of OA. The comparative literature on free-living PA following compartmental versus TKA is mixed.^44-46^ Some studies report comparable PA outcomes between procedures,^44^ while others show an improvement at early postoperative timepoints that is not sustained at longer-term follow-up.^45,46^ In the ACTION behavioural intervention trial, Hoorntje et al^44^ reported no difference in active time between procedures at six months. In contrast, Christensen et al^45^ in a large smartwatch-based study, reported significantly higher step count after UKA than TKA at one and three months, though this difference was not sustained at one year. This aligns with the findings of Theus-Steinmann et al^46^ who, in their retrospective study comparing UKA and TKA, reported that UKA was associated with a faster return of step count to preoperative levels. In the present study, Bi-UKA patients showed numerically higher PAEE and MVPA compared with TKA at one year (Figure 2, Table III), although differences in PA did not reach statistical significance at any timepoint. Overall, it is difficult to reach firm conclusions about the effect of compartmental arthroplasty on free-living PA. Existing comparative studies, of which there are few, differ in the types of monitoring device, the wear locations, and the outcome measures. Methodological heterogeneity is hence a concern when interpreting findings. Importantly, most studies were not powered a priori to detect between-procedure differences in PA. Adequately powered studies using standardized accelerometry methodology are needed to further explore this topic.

Bi-UKA is a cruciate-sparing, tissue-preserving alternative to TKA. Cadaveric studies indicate that Bi-UKA may preserve more physiological knee biomechanics than TKA.^47^ Clinical studies also report favourable gait outcomes after Bi-UKA.^8,10,48^ Most recently, in a matched study of 22 TKA, 22 Bi-UKA, and 24 healthy participants, Garner et al^10^ reported gait characteristics closer to healthy controls after Bi-UKA than TKA.^10^ Bi-UKA participants showed greater step length, nearer-normal cadence, and nearer-normal vertical ground reaction forces during mid-stance and maximum weight acceptance than TKA. On this basis, the primary hypothesis of the TRUCK trial was that a greater proportion of Bi-UKA patients would achieve a biphasic sagittal knee moment pattern during gait, compared with TKA.^22^ This pattern is typical of healthy knees and can be used as a marker of physiological gait.^22^ However, differences in the proportion of patients achieving biphasic gait at one or two years did not reach statistical significance.^22,41^ Therefore, although prior studies have reported potential gait advantages associated with Bi-UKA, such improvements were not clearly observed in our trial cohort. In this context, the absence of a statistical difference in PA outcomes is perhaps unsurprising.

In both the present analysis and the wider trial cohort,^41^ patient-reported outcomes improved comparably between TKA and Bi-UKA, suggesting similar satisfaction from surgery.^49^ Given similar improvement between groups in patient-reported symptoms, the lack of a difference in PA is plausible. We explored the relationship between PROMs and PA, though it should be noted that the trial was not powered to detect such relationships and was underpowered for PROMs in general.^40,41^ Correlations between PROMs and objective PA were inconsistent and were not observed in either group at two years. Similar findings have been reported in other knee arthroplasty populations. In a randomized trial comparing robotic and manual TKA, Clement et al^50^ reported no significant correlations between improvements in PROMs and objective PA. Frimpong et al^51^ similarly found no association between changes in PROMs and activity behaviours in a cohort of 43 patients undergoing TKA. These findings suggest that changes in objective PA may not necessarily parallel improvements in PROMs. As such, postoperative activity may not be determined by symptomatic relief alone. It is plausible that additional factors not measured in the present study, such as behavioural or psychosocial influences, may influence PA after knee arthroplasty. The impact of these non-surgical determinants within the present trial cohort is unknown.

While both groups showed similar postoperative PROMs and objective PA profiles, it is also unclear whether the procedures differ in physical capacity – the ability to perform activity under maximal effort. This aspect may represent the domain in which the purported kinematic advantages of Bi-UKA confers greatest benefit. Notably, Garner et al^10^ reported a 20% greater top walking speed, a maximal effort metric, following Bi-UKA compared with TKA on an instrumented treadmill. Other, more accessible capacity-based tests, such as the six-minute walk test, in which participants are asked to walk as far as possible in six minutes, have shown sensitivity to PA capacity gains after knee arthroplasty, and could help distinguish tissue-preserving procedures such as Bi-UKA.^52^ However, evidence specifically examining physical capacity following Bi-UKA remains limited. It may be that, within our trial cohort, Bi-UKA patients were able to perform more activity, but did not perform more activity in a free-living environment.

Given that knee arthroplasty alone may not be sufficient to enhance free-living PA, targeted rehabilitation and behavioural interventions could be of benefit. In a recent systematic review of 23 RCTs (n = 1,265), d’Unienville et al^53^ found that structured PA interventions yielded significant improvements in PA volume following TKA, though effects on MVPA and ST were small. Interventions that incorporated behaviour-change techniques, such as goal setting and counselling, achieved the greatest gains, particularly when initiated within two weeks of surgery.^53^ This represents a potential area of study, as it is unclear whether Bi-UKA patients are more responsive to structured activity interventions than TKA patients.

There are currently no protocols or standardized measures for calculating PA within orthopaedic literature,^54^ and consensus on analyzing accelerometry data to classify PA intensity is sparse.^18^ This makes comparison of reported figures difficult. Current evidence heavily relies on step count as a measure of PA,^55^ which captures only ambulatory movement and can underestimate total activity volume.^55^ Step count is also prone to misclassifying movement,^56^ a shortcoming especially relevant in arthroplasty patients, who may have abnormal gait patterns.^57^ Our use of ENMO-derived activity provides a more robust and clinically relevant measure of PA. ENMO integrates acceleration across three axes, including both ambulatory and non-ambulatory movement, and demonstrates stronger correlations with total movement volume.^56^ This method provides a more nuanced and complete assessment of PA, compared to step count. Crucially, ENMO is directly linked to PAEE, a known predictor of cardiometabolic health and all-cause mortality risk. The use of ENMO is supported by other works in the arthroplasty PA field,^58^ and, to our knowledge, this is the first study to apply ENMO-derived processing in the context of knee arthroplasty. We advocate for similar accelerometry approaches in future work.

This study has several limitations. The primary outcome of the TRUCK trial was the proportion of patients with a biphasic knee moment during gait at one year following surgery, and the study was powered a priori for this gait endpoint. Consequently, this secondary analysis was not powered to detect small-to-moderate differences between groups in PA. The absence of significant differences in PA should therefore not be interpreted as equivalence. However, to our knowledge, TRUCK is the only randomized trial comparing TKA and Bi-UKA, and thus provides the best available comparative evidence of both procedures. Additionally, attrition bias may have influenced our PA findings. Of the cohort that underwent surgery, 88% completed one-year follow-up and 53% completed two-year follow-up. The reduced two-year follow-up rate, which was largely due to the COVID-19 pandemic,^41^ limits the robustness of our conclusions at this timepoint. Moreover, PA is influenced by numerous behavioural and social factors, such as motivation and connectedness,^59,60^ which were not controlled for in the present study. Future studies could incorporate validated questionnaires, such as the Self-Efficacy for Exercise scale, to model the influence of these psychosocial factors on PA following arthroplasty.^61^ Finally, we encountered similar logistical challenges to those described by Clement et al,^50^ who found that wear non-compliance and unusable files limited complete accelerometer datasets. Nonetheless, among patients who provided accelerometer recordings, our overall compliance rate of 80% was comparable to, if not greater than, that reported in similar studies.^50,62^ Despite these challenges, we used a robust ENMO-based accelerometry protocol that yielded detailed metrics of PA.

In summary, few prospective studies have reported accelerometer-derived PA after TKA, and to our knowledge, no study has reported accelerometer-derived PA following Bi-UKA. This secondary analysis of a RCT therefore addresses a key evidence gap. We found that differences in PA between TKA and Bi-UKA failed to reach statistical significance across two years. Descriptively, both procedures were associated with greater free-living activity at one and two years postoperatively compared to preoperative baseline, and can hence be regarded a success. Future adequately powered randomized trials incorporating accelerometry are required to further compare PA outcomes following Bi-UKA and TKA.

## Data Availability

The data that support the findings of this study are available from the corresponding author upon reasonable request.
